# Immunological surrogate endpoints of COVID-2019 vaccines: the evidence we have versus the evidence we need

**DOI:** 10.1038/s41392-021-00481-y

**Published:** 2021-02-02

**Authors:** Pengfei Jin, Jingxin Li, Hongxing Pan, Yanfei Wu, Fengcai Zhu

**Affiliations:** 1 Department of Vaccine Clinical Evaluation, Jiangsu Province Center for Disease Control and Prevention, Nanjing, China; 2 NHC Key laboratory of Enteric Pathogenic Microbiology, Nanjing, China; 3 Department of Epidemiology and Biostatistics, School of Public Health, Southeast University, Nanjing, China

**Keywords:** Vaccines, Vaccines

## Abstract

In response to the severe acute respiratory syndrome coronavirus-2 (SARS-CoV-2) pandemic, over 200 vaccine candidates against coronavirus disease 2019 (COVID-2019) are under development and currently moving forward at an unparalleled speed. The availability of surrogate endpoints would help to avoid large-scale filed efficacy trials and facilitate the approval of vaccine candidates, which is crucial to control COVID-19 pandemic. Several phase 3 efficacy trials of COVID-19 vaccine candidates are under way, which provide opportunities for the determination of COVID-19 correlates of protection. In this paper, we review current knowledge for existence of COVID-19 correlates of protection, methods for assessment of immune correlates of protection and issues related to COVID-19 correlates of protection.

## Introduction

In response to the severe acute respiratory syndrome coronavirus-2 (SARS-CoV-2) pandemic, over 200 vaccine candidates against coronavirus disease 2019 (COVID-2019) are under development and currently moving forward at an unparalleled speed.^1^ Among of them, preliminary results from phase 3 efficacy trials are encouraging, with more than 90% efficacy against COVID-19 diseases for the two mRNA vaccines (BNT162b2 and mRNA-1273), and 70.4% and 91.4% efficacy for ChAdOx1 and rAd26/rAd5 COVID-2019 vaccine, respectively.^2–5^ Evaluation of the vaccine efficacy based on efficacy trials capturing clinical disease and/or infection as endpoints is the most direct approach to show the protection of vaccine candidate.^6^ However, phase 3 efficacy trials are very costly and time-consuming, which involve more than thousands of individuals in risk of SARS-CoV-2 exposure in order to provide enough power to show the protective efficacy of vaccine over the placebo. In addition, in the settings of available COVID-19 vaccines approved for emergency use or licensed, it will face a question of ethics to design a randomized placebo-controlled trials for assessing the efficacy of sequent vaccine candidates.

Immune correlate of protection (CoP) is an immunological assay (either humoral or cellular immune response) that reliably predicts protection against disease or infection after vaccination or natural infection.^7,8^ A CoP is of great importance because it can be used as a surrogate endpoint assessing vaccine efficacy without directly observing clinical endpoints.^9,10^ Comparing with large-scale field efficacy trials, immunological trials would save more than 60% of time and over 80% of expenses. Table 1 gives the estimates of CoPs for some licensed vaccines,^11^ some of which have been used for the evaluation of vaccine candidates compared with licensed vaccines in immunological trials. The availability of surrogate endpoints help to avoid large-scale filed efficacy trials and facilitate getting vaccine candidates approved,^12,13^ which is crucial to control COVID-19 pandemic. In addition, a CoP could also provide a convenient way to evaluate the immunological level of a population, which is essential in public health strategies.

**Table 1:** Correlates of protection after vaccination^11^

Vaccine	Immune function	Protection level
Adenovirus	Nt Ab	1/4
Anthrax	Toxin Nt Ab, anti-PA IgG	1/3000, 10 μg/mL
Diphtheria	Toxin Nt Ab	0.01–0.1 IU/mL
Enterovirus 71	Nt Ab	1:16–1:32
H. *influenzae* conjugate	ELISA Ab	0.15 ng/mL
Hepatitis A	ELISA Ab	20 mIU/mL
Hepatitis B	ELISA Ab	10 mIU/mL
Influenza, inactivated	HI Ab Nt Ab	1/40 = 50% protection, 1/320 in children 1/40 = 50% protection
Japanese encephalitis	Nt Ab	1/10
Lyme	ELISA Ab	1400 U/mL
Malaria	ELISA Ab CD4 + T-cell	>10 U/mL
Measles	ELISA Ab	≥120 mIU/mL
Meningococcal	Bactericidal Ab	≥1/4
Pneumococcal, conjugated	ELISA Ab	0.35 μg/mL
Polio, inactivated/live	Nt Ab	≥1/8
Rabies	Nt Ab	≥0.5 IU
Rubella	ELISA Ab	≥10–15 IU/mL
Smallpox	Nt Ab	≥1/20–1/32
Tetanus	Toxin Nt Ab	0.01–0.1 IU/mL
Varicella	GP ELISA	≥ 5 U/mL
Yellow fever	Nt Ab	≥0.7 LNI

*Ab* antibodies, *ELISA* enzyme-linked immunosorbent assay, *GP* glycoprotein, *HI* hemagglutination inhibition, *LNI* log neutralization index, *Nt* neutralization

In this paper, we review current knowledge for existence of COVID-19 correlates of protection, methods to evaluate CoP in efficacy trials from experiences with other vaccines and issues related to COVID-19 correlates of protection.

## Evidence for existence of COVID-19 correlates of protection

### Protective immunity after SARS-CoV-2 natural and experimental infection

The roles of humoral and cellular immune responses being responsible for protective immunity against SARS-CoV-2 in humans have not been fully understood. Most studies available on protective immunity had been performed in animal models. In re-challenge models of rhesus macaques,^14,15^ SARS-CoV-2 infection resulted in the induction of neutralizing antibody (NAb) and cellular immune responses including S-specific CD4^+^ and CD8^+^ T cells responses, and provided near-complete protection against SARS-CoV-2 re-challenge. In latter adoptive transfer studies,^16^ it was demonstrated that adoptive transfer of purified IgG from convalescent macaques^15^ protected naïve recipient rhesus macaques against SARS-CoV-2 infection in a dose dependent fashion. In particular, pseudovirus NAb titers of approximately 500 fully protected and titers of approximately 50 partially protected macaques against SARS-CoV-2 infection. In addition, depletion of CD8 + T cells in convalescent macaques partially abrogated the protective efficacy against SARS-CoV-2 re-challenge in the setting of NAb titers <100, suggesting that cellular immune responses are likely critical for virologic control if NAb titers are near or below the threshold titer required for protection.

Since SARS-CoV-2 is a novel pathogen, pre-exiting antibodies are limited. Moreover, it is unknown whether SARS-CoV-2 infection effectively protects against re-exposure in humans. Relatively few studies have been done to assess protective immunity based on observational studies relating pre-exposure immune level to COVID-19 disease or SARS-CoV-2 infection. Encouragingly, the Seattle Boat Study provides the first direct evidence that NAbs protect against SARS-CoV-2 re-infection in humans.^17^ In this outbreak of SARS-CoV-2 on a fishing vessel with an attack rate of 85.2%, none of three crew members who had NAbs (1:174, 1:161 and 1:3082, respectively) prior to departure were infected, whereas103 of 117 individuals who were seronegative prior to departure were infected. The results implicated NAb as a correlate of protection in humans, but we are unable to speculate a potential protective immunity level form this study due to limited data of pre-exposure NAbs, thus, further studies correlating pre-exposure NAbs with protection are warranted.

SARS-CoV-2-specific memory T cells will likely prove critical for long-term immune protection against COVID-19 and preventing severe COVID-19. Convalescent individuals exhibited robust memory T cell response months after SARS-CoV-2 infection, even in the absence of detectable specific circulating antibodies against SARS-CoV-2.^18,19^ Pre-existing, cross-reactive T cells have the capacity for accelerating virus clearance with improved clinical outcomes.^20^ Grifoni et al.^21^ identified presence of circulating SARS-CoV-2 specific CD8^+^ T and CD4^+^ T cells in most of COVID-19 convalescent patients (70% and 100%, respectively). Spike- specific CD4^+^ T cell responses correlated well with the magnitude of the anti-spike IgG and IgA titers. Importantly, non-spike-specific CD4^+^ T cell responses against SARS-CoV-2 were detected in 40–60% of unexposed individuals, indicating that some potential for cross-reactive induced by common cold coronaviruses. In the subsequent study,^22^ this research team demonstrated a range of preexisting memory CD4 + T cells that react to SARS-CoV-2 epitopes actually cross-react with corresponding homologous sequences from many common cold coronaviruses HCoV-OC43, HCoV-229E, HCoV-NL63, or HCoV-HKU1. To identify functional and phenotypic landscape of SARS-CoV-2-specific T cell responses across the full spectrum of COVID-19. Sekine et al.^19^ systematically mapped T cell responses against SARS-CoV-2 in individuals with acute or convalescent COVID-19, exposed family members and unexposed individuals. The results showed that acute-phase SARS-CoV-2-specific T cells displayed a highly activated cytotoxic phenotype that correlated with various clinical markers of disease severity, whereas convalescent-phase SARS-CoV-2-specific T cells were polyfunctional and displayed a stem-like memory phenotype. Importantly, SARS-CoV-2-specific T cells were detectable in antibody-seronegative exposed family members and convalescent individuals with a history of asymptomatic and mild COVID-19. It remains to be determined whether robust memory T cell responses in the absence of detectable circulating antibodies can protect against severe forms of COVID-19 in humans. Results from experimental and natural infection studies indicate that SARS-CoV-2 infection can induce protective immunity against reinfection by eliciting NAbs, and cellular immunity is likely critical for virologic control and reducing severity of COVID-19 diseases.

### Protective immunity induced by COVID-19 vaccines

Most effective human vaccines work by generating antigen-specific functional antibodies,^23,24^ specifically NAbs, which block the entry of the virus into target host cells and prevent infection. Based on the understanding of the interaction of SARS-CoV-2 with host cell, and the immune response to SARS-CoV-2 after nature infection, SARS-CoV-2 Spike protein is identified as an antigenic target for the development of most investigational vaccines.^25^ Hence, the induction of NAbs against SARS-CoV-2 Spike protein is the primary goal of COVID-19 vaccine candidates.

Human challenge trials could accelerate the testing of COVID-19 vaccine candidates and provide rapid assessment for a protective titers of immune CoP by assessing the relationship between antibody titers induced by vaccination and protection post-challenge.^26,27^ Currently, it is controversial due to the severity and unknown long-term impacts of SARS-CoV-2 infection and concerns over ethical administration of such trials.^28,29^ In the absence of data for humans, the result of studies in non-human primates (NHPs) can offer some clues, and help to identify potential CoPs for COVID-19 vaccine candidates. The studies showed that COVID-19 vaccine candidates from different platforms provided protection form infection with SARS-CoV-2 in NHPs,^30–36^ three of which assessed potential immune correlates of protection by taking advantage of the variability in protective efficacy.^30,31,35^ The results showed that vaccine-elicited NAb titers correlated with protection against SARS-CoV-2 challenge, suggesting as a potential immune correlate of protection. The findings of DNA and Ad26 vaccine studies in NHPs showed that live virus NAb titers (based on microneutralization assay with 50% reduction in relative light units as readout) of approximately 100–250 (10^2^−10^2.4^) were required for complete protection against SARS-CoV-2 infection.^30,31^ Moreover, a logistic regression analysis showed that NAb combined with diverse Fc functional antibody responses improved correlation with protection, which suggests that NAbs have a primary role in protecting against SARS-CoV-2, supported by other binding and functional antibodies (i.e. antibody-dependent complement deposition (ADCD) and antibody-dependent monocyte cellular phagocytosis (ADCP)). By contrast, vaccine-elicited ELISPOT responses, and CD4^+^ and CD8^+^ intracellular cytokine staining responses, did not correlate with protection. These data suggest that NAb may be a potential CoP and NAb titers of 100-250 may be a protective antibody level against SARS-CoV-2 infection in NHPs, although this will need to be confirmed in vaccine efficacy studies in humans.

As we know, there are currently multiple efficacy trials of COVID-19 vaccine candidates under way (see Table 2). The results of these COVID-19 vaccine candidates in phase I/II trials have been shown to elicit levels of NAbs being equal to or higher than those observed in convalescent patients, and cellular immune responses.^37–46^ However, the results are difficult to compare due to different assays and readouts were used. Excitingly, the preliminary analysis demonstrated the efficacy of COVID-19 vaccine candidates, ranging from 70% to 95%.^2–5^ The promising results will contribute to identify COVID-19 correlates of protection on the basis of data collected from phase III efficacy trials.

**Table 2:** Clinical trials results of COVID-19 vaccine candidates ongoing in phase III efficacy trials

Vaccine developer	Platform	Target dose and schedule in phase III trial	Preliminary vaccine efficacy against COVID-19	Immunogenicity of target dose and schedule in phase I/II trial	
				Neutralizing antibody titers	T cell response
Moderna^3,37,38^ (mRNA-1273)	mRNA expressing spike protein	100 μg (Day 0, 28)	94.1%	1:654 (18-55 yeas)^a^ 1:878 (56-70 years^a^ 1:317 (≥71 years)^a^	CD4 Th1 cell responses and low CD8 T cell responses
Pfizer/BioNTech SE^2,39^ (BNT162b2)	mRNA expressing spike protein	30 μg (Day 0, 21)	95.0%	1:163 (18-55 years)^b^ 1:206 (65-85 years)^b^	ND
AatraZeneca/University of Oxfors^4,40^ (ChAdOx1)	Non-replicating chimpanzee adenoviral vector expressing spike protein	Low dose (2.55 × 10^10^VP)/ Standard dose (5 × 10^10^VP) (Day 0, 28)	70.4% (Low-standard dose: 90.0%; Standard-standard dose: 62.1%)	Low-low dose/ Standard- Standard dose 1:161/1:193 (18-55 years)^c^ 1:143/1:144 (56-69 years)^c^ 1:150/1:161 (≥70 years)^c^	IFNγ-ELISpot T-cell responses
Gamaleya Center^5,41^ (rAd26/rAd5)	Non-replicating recombinant adenoviral type26 and type5 expressing spike protein	10 × 10^10^VP (Day 0, 21)	91.4%	1:46-1:49 (18-60 yeas)^d^	CD4 Th1 and CD8 T cell responses
CanSino Biological Inc./Beijing Institute of Biotechnology^42^	Non-replicating recombinant adenoviral type5 expressing spike protein	5 × 10^10^VP (Day 0)	ND	1:21.2 (18-44 years)^e^ 1:17.8 (45-54 years)^e^ 1:9.6 (≥55 years)^e^	IFNγ-ELISpot T-cell responses
Janssen^43^ (Ad26.COV2.S)	Non-replicating recombinant adenoviral type26 expressing spike protein	5 × 10^10^VP (Day 0)	ND	1:214 (18-55 years)^f^ 1;196 (≥65 years) f	CD4 Th1 and CD8 T cell responses
Sinovac^44^ (CoronaVac)	Inactivated	3ug (Day 0, 14)	ND	1:27.6 (18-59 yeas)^g^	ND
Beijing Institute of Biological Products^45^ (BBIBP-CorV)	Inactivated	4ug (Day 0, 21)	ND	1:218.9 (18-59 yeas)^h^	ND
Novavax^46^ (NVX-Cov2373)	Recombinant SARS-CoV-2 glycoprotein nanoparticle vaccine adjuvanted with Matrix-M	5 μg SARS-CoV-2 rS + 50 μg Matrix-M1 adjuvant (Day 0, 21)	ND	1:3906.3 (18-79 years)^i^	CD4 Th1 cell responses

^a^Based on live virus 80% plaque-reduction neutralization testing (PRNT80) assay

^b^Based on wild-type virus microneutralization assay (MNA) with an inhibitory concentration of 50%, with relative light units as readout

^c^Based on wild-type virus MNA with an inhibitory concentration of 80%

^d^Based on wild-type virus MNA with a 50% tissue culture infective dose of 100 (100TCID50)

^e^Based on wild-type virus MNA with an inhibitory concentration of 50%, with Cytopathic effect (CPE) as readout

^f^Based on wild-type virus MNA with an inhibitory concentration of 50%

^g^Based on wild-type virus MNA with Cytopathic effect (CPE) as readout

^h^Based on live virus 50% plaque-reduction neutralization testing (PRNT50) assay

^i^Based on wild-type virus MNA with an inhibitory concentration of 99%

*ND* not determined

## Methods to evaluate Cop in efficacy trials from experiences with other vaccines

Establishing a cutoff level of immune CoP is the ultimate objective of investigating the immunological CoP. Historically, a variety of study designs and methods have been utilized to evaluate protective immunological level induced by vaccine, which are informative to identifying COVID-19 CoP, including randomized control trials with clinical endpoints, human challenge studies, passive immunization studies, observational studies, the extrapolation of animal studies and so on. Randomized control trials (RCT) provide the ideal context to assess the relationship between vaccine, CoP and clinical endpoints. In a randomized placebo-controlled trial, eligible participants are randomly assigned to receive either vaccine candidate or placebo, followed by collecting blood sample from all participant at the peak of vaccine-induced immune response to assess CoP, with observed clinical endpoints during the surveillance period. Three methods were currently applied to assess a cutoff level of CoP based on numerous vaccine clinical studies, including threshold method, continuous method based on case-cohort study, and receiver operating characteristic (ROC) curve method by using case-control design.

The threshold method for finding the cutoff level of an immunological assay that quantitatively predicts the efficacy of the vaccine was developed in the 7-valent pneumococcal conjugate vaccine efficacy trial.^47,48^ In the threshold method, it assumes a step function between immunological CoP and the probability of disease that individuals with above than the threshold level of CoP are fully protected and blew which individuals remain fully susceptible. The threshold method uses the proportion of vaccinated and unvaccinated individuals below specified thresholds to estimate vaccine efficacy (VE):

$$\begin{array}{l}{\mathrm{VE}} = 1 - \frac{{{\mathrm{Pr}}[disease(vaccinated)]}}{{{\mathrm{Pr}}[disease(unvaccinated)]}}\\\quad\ \ = 1 - \frac{{{\mathrm{Pr}}[individuals\,less\,than\,the\,cutoff(vaccinated)]}}{{{\mathrm{Pr}}[individuals\,less\,than\,the\,cutoff(unvaccinated)]}}{.}\end{array}$$

In other words, the relative risk of disease equals the relative rate of individuals with immune CoP less than the cutoff, and the predicted vaccine efficacy estimates for various cutoffs were compared with the observed vaccine efficacy. If the predicted vaccine efficacy for a given cutoff is consistent with the observed vaccine efficacy, this cutoff may be the valid cutoff for correlate of protection. Data from three efficacy trials of pneumococcal conjugate vaccines were aggregated to derive a protective threshold of 0.35 μg/mL anti-pneumococcal capsular polysaccharide antibody, which corresponds to the observed VE of 93.0%.^49^ The threshold concentration of 0.35 μg/mL has been recommended by the WHO Working Group as applicable on a global basis for assessing the efficacy of future pneumococcal conjugate vaccines, without the need for further large-scaled efficacy trials.

A limitation of the threshold method is that the relationship between assay values and the occurrence of disease below the threshold level is usually not specified, and individuals with low assay values might not develop disease because of not being exposed.^50^ In addition, individuals with assay values above the chosen threshold will occasionally develop disease.^49^ Theoretically, the relationship between risk of disease and immune response marker can be modeled as a continuous function. To address this problem, Dunning^50^ proposed the scaled logit model for calculating titer-specific rates of disease, which includes the continuous relationship between levels of the immune marker and protection in which exposure is modeled explicitly. In phase III efficacy trials of influenza and enterovirus 71vaccines, the continuous method has been applied to assess respective CoP.^51–54^. The risk of disease could be highly influenced by the probability of exposure to an infectious pathogen. Currently, HI ≥ 40 corresponding to 50% protection from infection was recommended as surrogate of protection to evaluate the efficacy of new influenza vaccines. However, it needs higher HI titer to provide 50% protection against influenza illness in children and older individuals.^51,52^ Therefore, it is necessary to appropriately improve the protection level in high-risk population (i.e., old individuals and those with underlying health conditions in COVID-19 pandemic) or high prevalence areas. Meanwhile, targeting a high level of protection against the risk of infection can reduce pathogen circulation in population and provide the indirect effects, which has important public health implications.

In the efficacy trials of influenza and enterovirus 71 vaccines, receiver operating characteristic (ROC) curve was also applied to determine the cutoff level of immunological CoP based on case-control designs.^55–58^ In the ROC curve, sensitivity was defined as the proportion of individuals with a post-vaccination titer below the cutoff value among those with confirmed clinical disease; specificity was defined as the proportion of individuals with a post-vaccination titer equal to or greater than the cutoff value among those without confirmed clinical disease. The best cutoff value for the immunological CoP could best separates the subjects who were protected from those who were not, at which the sum of the specificity and sensitivity is maximum. In the ROC curve analysis, the Youden index gives the same weight to both sensitivity and specificity, which separates the protected and non-protected individuals. However, the rates of infectious disease among individuals with low titers may be strongly associated with the chance of exposure and disease prevalence, and individuals who were not infected are a mixture of individuals who were protected and those who were unprotected but also unexposed. Therefore, false negatives are likely to occur and sensitivity should determine the cut-off value, and it is necessary to improve sensitivity to reduce false negatives in accordance with the exposure level.

Three methods with different interpretations have been developed to explore the surrogate protection for vaccine clinical trials in terms of absolute and relative CoP,^9,11^ we need to select appropriate methods according to the characteristic of vaccine candidates, different study designs and data collected in ongoing phase III efficacy trials. Therefore, using a CoP in the evaluation of vaccine clinical trials, we should take consideration of other issues related to immunological CoP, including the definition of clinical endpoint, type of vaccine candidates, the target population, exposure intensity, as well as the generalizability of the results to other settings.

## Opportunities and challenges for COVID-19 correlates of protection

Over 200 COVID-19 vaccine candidates, based on several different platforms, are currently is moving forward at an unparalleled speed, which will provide opportunities to identify COVID-19 correlates of protection. Meanwhile, breadth of global response presents challenges to identification of correlates of protection. It is essential to specify standardized, quantifiable endpoints against which the vaccine is expected to protect, since it may be variable the relationship between CoP and different endpoints. An efficacious COVID-19 vaccine could reduce the likelihood of symptomatic infection, severity of disease, or degree of transmission. In general, NAb and T cellular immunity are needed to protect against severe disease, and mucosal immune response and higher NAb titers could be required to prevent transmission of pathogens.^9^

Notably, vaccine-induced protective immune response may be various for vaccine candidates from different platforms. Cellular immune responses have been described in response to infection and are likely to be an important component of a protective immune response. Among COVID-19 vaccine candidates onging in phase III efficacy trials, vectors vaccines, mRNA vaccines and recombinant protein vaccines are able to elicit T-cell responses,^59^ but it is not clear whether the relative contribution to efficacy of T-cell responses is consistent for different vaccine candidates, which may have an effect on the application of a universal CoP.

NHPs studies provided evidence for antibody-mediated protection against SARS-CoV-2 infection, in which timepoint of challenge is at day 14 or 28 post-vaccination (at the peak point of antibody production), so a key question concerns the durability of antibody responses induced by COVID-19 vaccines. Currently, the immune persistence of COVID-19 vaccines is yet to be known. Several recent studies investigated the durability of the antibody responses (binding antibody and neutralizing antibody) in SARS-CoV-2 infection, but opposite results were presented.^60^ As we known, antibody titers pre-exposure is most correlated with disease and infection. NAb titers correlating with clinical endpoints, obtained from clinical trials, may not represent true protective immunity level if there is a “rapid decay” of NAb titers after vaccination.

Measurement of vaccine-induced immune markers is a key component for establishing COVID-19 CoP. A variety of serological assays have been developed, including live virus neutralizing assays in different formats, pseudo virus neutralizing assays using different agents and ELISA assays using different target binding antigens. Comparing immune response against different vaccine candidates is challenging due to lack of standardization among different assays and different testing laboratories. WHO Collaborating Center for Biological Standardization and National Institute for Biological Standards and Control (NIBSC) in UK are developing the WHO international standard and reference panel for anti-SARS-CoV-2 antibody.^61^

Efficacy trials have the advantage of identifying the association vaccination, immune markers and protection. However, the enormous resources required to conduct such studies may limit the number that can be done, including the actual number of diseases, the number, quality of biological samples collected, and the number of collection time points.^62^ For example, to determine the immune correlate of protection for inactivated enterovirus 71 vaccine, blood samples from participants of approximately 20,000 at day (before the first dose) and day 56 (28 days after second dose) were collected in two efficacy trials.^57,58^ Differences between vaccines, immune responses and clinical endpoints, and the standardization of assessing immune markers as well as resource needs, present challenges for COVID-19 CoP, so much work remains to be done.

## Concluding remarks and perspectives

In conclusion, current NHPs studies indicate that NAb targeting spike protein is a potential and practical CoP supported by other Fc functional antibody responses, and cellular immunity is likely critical for virologic control and reducing severity of COVID-19 diseases. NHPs challenge studies showed that live virus NAb titers of 100-250, based on microneutralization assay with 50% reduction in relative light units as readout, may be a protective immune level against SARS-CoV-2 infection in NHPs. However, there may be important differences between SARS-CoV-2 infection in macaques and human beings, including racial differences, intensity and timepoint of exposure and other unknown factors, so protective immune level obtained in animal studies would need validation in clinical trials.

With the development of international standards assessing immune markers and the cooperation of international multicenter clinical trials, it will provide opportunities for the determination of COVID-19 correlates of protection.
